# Replacing conventional decontamination of hatching eggs with a natural defense strategy based on antimicrobial, volatile pyrazines

**DOI:** 10.1038/s41598-017-13579-7

**Published:** 2017-10-16

**Authors:** Peter Kusstatscher, Tomislav Cernava, Stefan Liebminger, Gabriele Berg

**Affiliations:** 1 ACIB GmbH, Petersgasse 14, 8010 Graz, Austria; 2 Institute of Environmental Biotechnology, Graz University of Technology, Petersgasse 12, 8010 Graz, Austria; 3 Roombiotic GmbH, Petersgasse 12, 8010 Graz, Austria

## Abstract

The treatment of hatching eggs relies on classic yet environmentally harmful decontamination methods such as formaldehyde fumigation. We evaluated bacteria-derived volatiles as a replacement within a fundamentally novel approach based on volatile organic compounds (VOCs), which are naturally involved in microbial communication and antagonism due to their high antimicrobial efficiency. Pyrazine (5-isobutyl-2,3-dimethylpyrazine) was applied passively and actively in prototypes of a pre-industry-scale utilization. Altogether, pyrazine decontamination rates of up to 99.6% were observed, which is comparable to formaldehyde fumigation. While active evaporation was highly efficient in all experiments, passive treatment showed reducing effects in two of four tested groups only. These results were confirmed by visualization using LIVE/DEAD staining microscopy. The natural egg shell microbiome was characterized by an unexpected bacterial diversity of *Pseudomonadales, Enterobacteriales, Sphingomonadales, Streptophyta*, *Burkholderiales*, *Actinomycetales*, *Xanthomonadales*, *Rhizobiales*, *Bacillales*, *Clostridiales*, *Lactobacillales*, and *Flavobacteriales* members. Interestingly, we found that especially low pyrazine concentrations lead to a microbiome shift, which can be explained by varying antimicrobial effects on different microorganisms. *Micrococcus* spp., which are linked to embryonic death and reduced hatchability, was found to be highly sensitive to pyrazines. Taken together, pyrazine application was shown to be a promising, environmentally friendly alternative for fumigation treatments of hatchery eggs.

## Introduction

Conventional decontamination of hatchery eggs is primarily achieved by formaldehyde fumigation of the eggshells. This treatment ensures a high efficiency in the reduction of potentially pathogenic microorganisms in industrial-scale hatcheries^1,2^. However, formaldehyde is also linked to adverse health effects and classified as a pollutant^3^. It has been known for a long time that formaldehyde-induced pathogenesis including bronchitis, pneumonia and cancer^4,5^. Due to these harmful health effects, efficient alternatives are needed that can replace formaldehyde in industry-scale hatcheries. The ongoing approach to find feasible replacements is exacerbated by the high diversity of occurring pathogens^6,7^. *Pseudomonas*, *Escherichia*, *Salmonella* and *Mycoplasma* are causing substantial damage in industrial hatcheries, including increased early embryonic mortality or egg yolk infection^6,8^. Especially the formation of resistances in relevant pathogens against conventional disinfectants aggravates the implementation of new pathogen controlling precautions in this sector^9^.

Defense against pathogens is one of the major functions of the host-associated microbiota^10^. The mode of action of pathogen suppression incudes competition and is supported by the production of antimicrobials including volatile organic compounds (VOC)^11^. Recently, VOCs produced by plant-associated microorganisms that can inhibit a broad range of human and plant pathogens were identified^12,13^. While studying plant-associated *Paenibacillus* isolates – especially various strains of *Paenibacillus polymyxa –* the antimicrobial activity could not be explained solely by known antimicrobial compounds that were present in the headspace^14^. In this context, alkyl-substituted pyrazines were detected as determinants of the strong antimicrobial effects and confirmed as the main antimicrobial VOCs in the headspace of plant-associated *P. polymyxa* isolates^14^. These plant-associated isolate demonstrated high biocontrol potential due to their pronounced antagonistic activity against devastating plant pathogens^15^. Interestingly, the same isolate also inhibited potential human pathogens when they were exposed to its volatiles^14^. The overall collected data suggested a broad range of efficiency, thus mimicking its bioactive volatilome is a promising strategy to control diverse microbial communities. Based on these findings we further on utilized 5-isobutyl-2,3-dimethylpyrazine as a model pyrazine, which has a similar antimicrobial efficiency to the pyrazine mixture that is emitted by *P. polymyxa* GnDWu39^15^.

Due to the high vapor pressure of pyrazines at room temperature, the compounds volatilize easily and can be applied for the decontamination of microstructures. Similar advantages are also observed in combination with hydrogen peroxide treatments^16^. Here, the low stability and high reactivity of the disinfectant are often disadvantageous. In contrast, the properties of pyrazines are facilitative not only in the decontamination of surfaces, but also be applied on decontamination strategies in the food industry and related sub-sectors. Protective atmospheres as well as contamination control in processed food products are possible applications due to the advantageous properties of the bioactive volatiles. This study’s focus was on the evaluation of a decontamination strategy of hatching eggs using 5-isobutyl-2,3-dimethylpyrazine as an antimicrobial active model pyrazine. The round surface of eggs coupled with their fragility makes liquid application of decontaminating agents less efficient^6,8^. For this purpose the evaporating characteristic of pyrazines was implemented in two different application strategies. These sealed container evaluations of application methods served as early prototypes of a pre-industry-scale utilization of the novel decontaminant. The utilized pyrazine derivative was either evaporated passively or actively for an enhanced saturation of the headspace of a sealed container. The efficiency of the treatment was evaluated using growth dependent as well as independent methods. In addition, amplicon sequencing of the 16 S rRNA gene fragment of the total community DNA obtained from egg shells was included to study the impact on the entire egg microbiome. This study provides a first assessment of the promising potential of alkylated pyrazines with strong antimicrobial effects for further utilization in industrial applications.

## Results

### Microbial load was significantly reduced after pyrazine treatments

**Figure 1:**
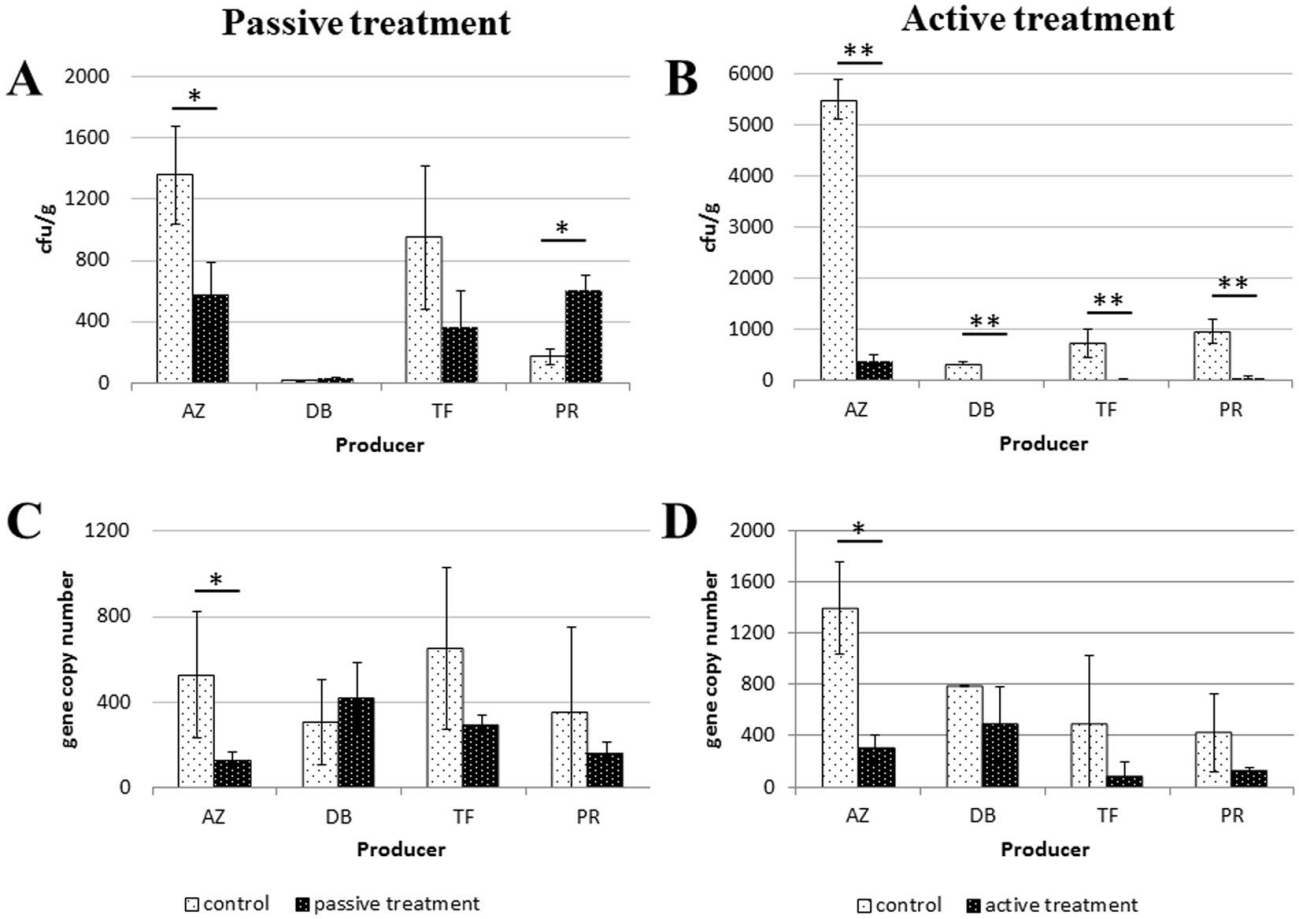
Cultivation-based and molecular quantification of bacteria on the eggshell surface. Eggs from different producers were treated with passive (**A,C**) and active (**B,D**) fumigation in combination with a highly bioactive diazine derivative. Bars represent cfu and gene copy number counts respectively. Significance levels were determined with Student’s t-test. *p < 0.05; **p < 0.01.

The efficacy of two treatment strategies was evaluated using cultivation dependent methods analyzing colony forming units (CFUs) counts (Fig. 1A and B). Higher treatment efficiency is shown by lower CFU counts indicating a lower microbial load. Active evaporation was highly efficient in all samples and led to statistically significant CFU reductions (Fig. 1B), whereas passive treatment showed reducing effects in two of four tested groups (AZ and TF). The CFU counts were increasing in the other samples (DB and PR; Fig. 1A). Highest contamination levels were found in samples from farm-gate sale egg samples (AZ). Reduction rates were calculated from the CFU counts to evaluate the treatment efficacy. Passive treatment showed reduction rates from −62.2% (TF) to −57.4% (AZ). However, also increasing effects from +77.8% (DB) to +249.6% (PR) were observed for passively treated samples. In contrast, active treatment always led to reduced viability in the range between −93.1% (AZ) and −99.6% (DB).

RT qPCR-quantification, analyzing gene copy numbers in DNA extracts from the samples, confirmed the effects shown in cultivation dependent methods (Fig. 1C and D). Passive treatment had reducing (AZ, TF and PR) and increasing (DB) effects whereas active treatment showed reducing effects in all samples. The gene copy numbers g^−1^ eggshell of the bacterial marker gene ranged between 250.7 (AZ; control: 940.8) and 546.1 (DB) for the passively treated samples. In the active treatments the range was covered between 203.1 (TF; control: 617) and 610 (DB). However, overall lower significance was found for the observed reductions on treated samples.

### Treatment-induced shifts in highly diversified microbial communities

**Figure 2:**
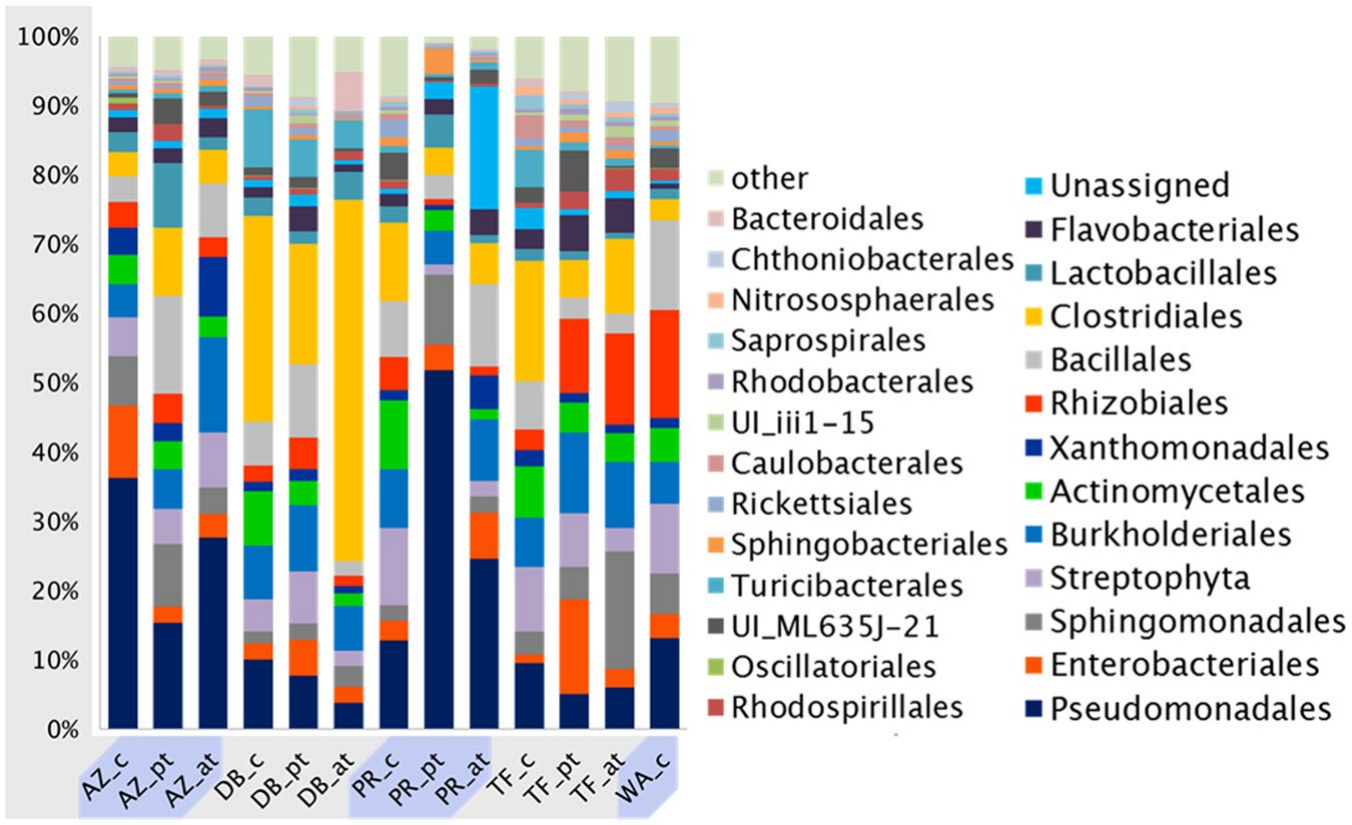
Observed diversity in all samples (order level). Eggshells were fumigated with bioactive pyrazines in two specific treatments. Community DNA extractions from the outer surface were amplified with bacteria-specific primers and subjected to amplicon sequencing. Each bar includes the mean abundance from four combined samples. **c**: **c**ontrol; **pt**: **p**assive **t**reatment; **at**: **a**ctive **t**reatment; WA_c: non-template control.

Bioinformatic evaluation of the 16 S rRNA amplicon sequencing data indicated high diversity in the egg samples. High abundances were found for the orders *Pseudomonadales* (4–52%), *Enterobacteriales* (1–10%), *Sphingomonadales* (2–17%), *Streptophyta* (1–11%), *Burkholderiales* (5–12%), *Actinomycetales* (1–8%), *Xanthomonadales* (1–9%), *Rhizobiales* (1–16%), *Bacillales* (2–14%), *Clostridiales* (3–52%), *Lactobacillales* (1–9%), and *Flavobacteriales* (1–4%). Notably, *Clostridiales* were highly abundant in the DB samples (17–52%) as well as *Pseudomonadales* in the AZ samples (15–36%) and PR samples (12–52%; Fig. 2). However, no global correlation of treatment and changes in OTU abundance was found on order level. Similar community structures on order level for samples from the same producer can be seen in Fig. 2 (AZ, DB). Moreover, the community structure of control samples (dH_2_O instead of template DNA) was found to have analogous distributions as the sample controls (Fig. 2A, WA_c).

Treatment efficiency on phylum level showed an increasing *Proteobacteria* proportion following passive treatment, indicating a community shift between the treatments (Figure S3). *Actinobacteria* are reduced during both treatments (from 8% in the control to 4%). The fraction of unassigned OTUs increased during active treatment (from 2% in the control up to 8%).

### Significant structural community changes on species level

**Figure 3:**
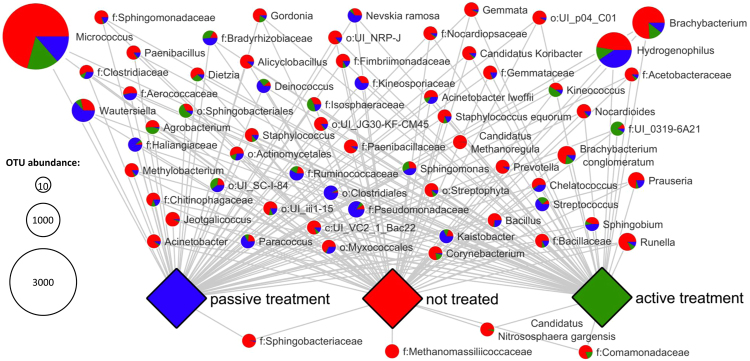
Abundance and proportions of OTUs on treated and untreated samples. Two distinct treatments and untreated samples were compared in terms of pyrazine-induced changes of specific taxon abundance. The node size correlates to the total abundance. Pie charts indicate the fractions found in distinct samples.

Deepening bioinformatic analyses were employed to identify specific OTUs that were significantly affected in their abundance following the pyrazine treatment. The corresponding group significance analysis for the identification of statistically significant differences on species level of the treatment methods was visualized using Cytoscape 3.4.0 (Fig. 3). Most of the OTUs were present in both treatments as well as the control. In contrast, only a minor fraction of OTUs was found exclusively in one treatment or the control. The families of *Methanomassiliicoccaceae* (Archaea) and *Sphingobacteriaceae* disappeared completely following pyrazine treatments. Furthermore, the majority of the nodes (80 out of 100), which represent distinct OTUs, show the highest fraction for untreated samples. This correlates with a high occurrence of the respective microorganism the controls. In contrast, 20 of the nodes indicate an increasing fraction of OTUs induced by a specific treatment. A total of 6 OTUs were increased by active treatment and 14 OTUs by passive treatment. *Clostridiales, Paracoccus, Nevskia ramose, Sphingobium*, UI_SC-I-84, *Bradyrhizobiaceae, Ruminococcaceae, Kineosporiaceae, Paracoccus, Kaistobacter, Deinococcus, Clostridiales, Streptococcus* as well as *Pseudomonadaceae* are the most prominent examples highly increased by passive treatment. *Kineococcus, Sphingobacteriales, Streptophyta, Chitinophagaceae, UI_0319–6A21* and *Isosphaeraceae* were found to be increased by active treatment.

### Treatment efficiency visualized by differential staining

**Figure 4:**
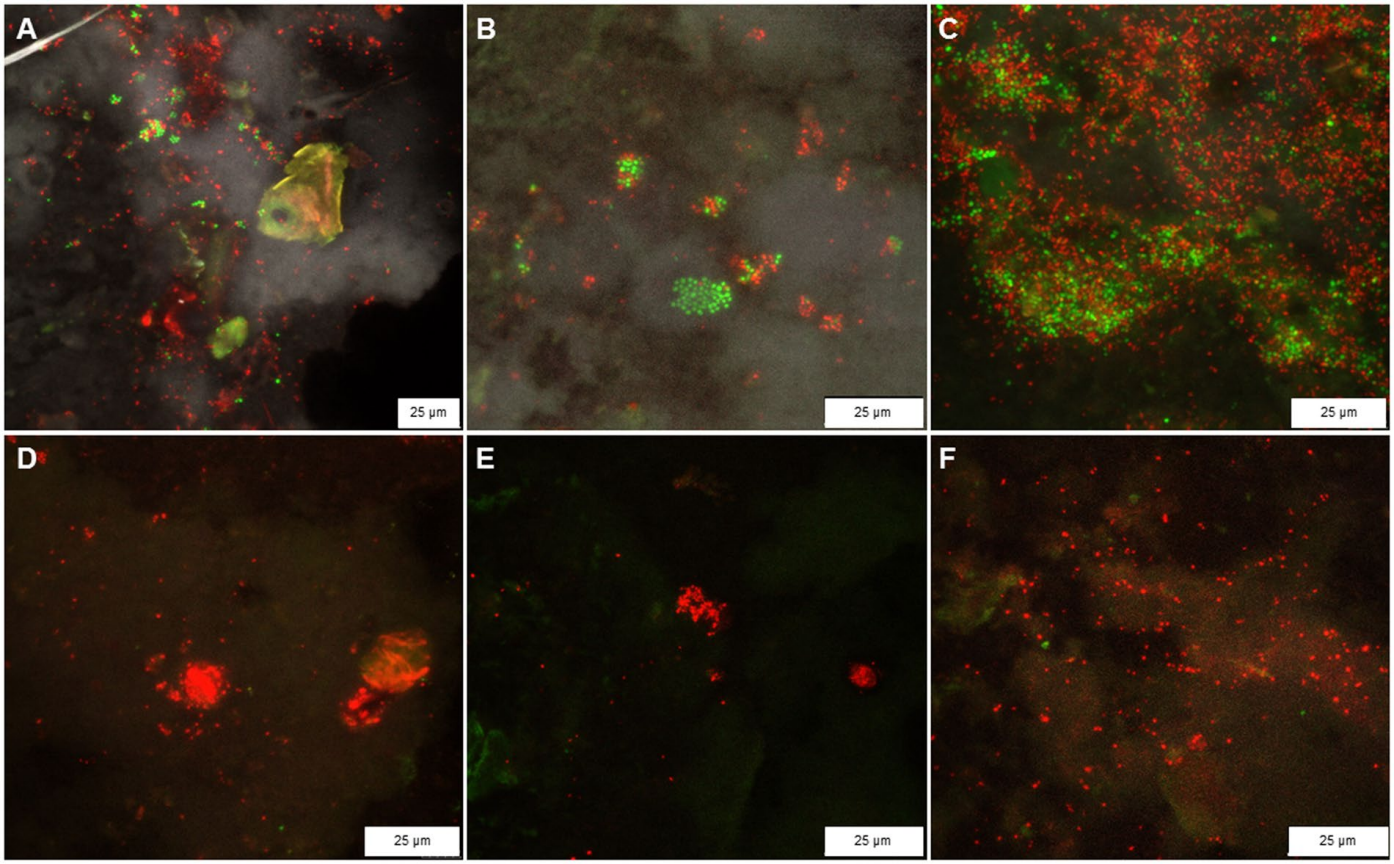
LIVE/DEAD staining of bacteria on egg shells. The visualization of living and dead bacteria on eggshells allows additional assessments of the treatment efficiency. Green: living cells; red: dead cells. (**A**–**C**) shells of untreated eggs; (**D**–**F**) shells of diazine-treated eggs.

LIVE/DEAD-stained bacteria located on eggshell surfaces were visualized using confocal laser scanning microscopy (CLSM). Micrographs showing differentially stained bacterial eggshell colonizers are shown in Fig. 4. Active treated egg shells (micrographs D to F) in comparison to control egg shells (micrographs A to C) showed reduced bacterial counts. A high proportion of living bacteria were found on control egg shells, whereas no living cells were found on treated samples. The obtained micrographs indicate similar reduction rates to such determined with cultivation-dependent methods.

## Discussion

### Pyrazine treatment as a new decontamination strategy for hatching eggs

In this study, a new decontamination technology for chicken eggs was evaluated to overcome risks related to pathogen propagation in hatcheries. Hatching eggs are affected by microbial contaminations and are usually treated with formaldehyde to guarantee a safe chicken development^17^. Typical microbial contaminations in large-scale hatcheries are caused by *Salmonella, Pseudomonas, Micrococcus*, or *Escherichia* ^18,19^. Precisely because of, the harmful effects of formaldehyde that are known for a long time, hatcheries would benefit from new, more environmental friendly decontamination methods^4,5^. The widespread availability and the sufficient decontamination efficiency are the main drivers for the application of formaldehyde. In fact, the fumigation of eggshells with formaldehyde leads to a reduction of 99.85% for all present bacteria^1,2^. In our first assessment, pyrazine treatment was shown to have comparable decontamination rates of 99.6% in comparison to formaldehyde fumigation, when an adjusted application method was used.

Both, cultivation-dependent and RT qPCR analysis demonstrated the efficiency of 5-isobutyl-2,3-dimethylpyrazine in the decontamination approaches. While passive treatment showed a reduced efficacy, active treatment was shown to substantially decrease the total number of bacteria in all samples with reduction rates up to 99.6%. This is most likely due to the higher pyrazine concentrations in the headspace of the containers due to the enhanced volatilization. Moreover, we observed high standard deviations for the control samples. This can be explained by the inconsistent amounts of stable-derived residues on the egg surface. RT qPCR showed generally lower reduction rates, which can be caused by inefficient or incomplete PMA reactions. The efficacy of PMA-based DNA blockage in dead cells is directly linked to cell membrane intactness, whereas other concepts of molecular viability testing rely on metabolic functions^20,21^. Although the reduction rates obtained with the molecular approach were lower, the general results from cultivation-dependent methods were confirmed. Such tendencies were also visualized with LIVE/DEAD-staining micrographs.

The utilization of microbial volatiles in industrial applications harbors the chance to remove biological contaminants with the weapons of their natural antagonists. It was often observed that highly active antagonists inhabit various densely colonized niches in nature and that they can shield off pathogen attacks. This especially applies for plant-associated habitats that often provide shelter for several hundreds of different microorganism species^22,23^. *Paenibacillus polymyxa* GnDWu39 that was isolated from *Cucurbita pepo* var. styriaca represents such highly antagonistic colonizer^15^. It was also the first isolate that provided evidence for the broad antimicrobial effects of distinct alkylated pyrazines^14^.

Pyrazines are widely spread in nature and primarily known for their characteristic scent^24,25^. Plants serve as natural source for pyrazines, while bacteria are often used for the industrial production of specific pyrazine derivatives^26^. The aroma of several vegetables such as beetroots, silver beets, carrots, broccoli, cucumber, asparagus and lettuce is mainly due to various pyrazine compounds^27^. Moreover, the Maillard reaction during cooking processes leads to pyrazine formation in heated food^25^. Due to their widespread occurrence and good availability on the chemicals market, bioactive pyrazines are promising substances for future decontamination approaches. More importantly, the so far evaluated derivatives of alkylated pyrazines are regarded as safe for human consumption at certain intake levels^28^.

### Treatment efficiency and pyrazine induced structural community changes

This study evaluated the influence of volatile antimicrobial pyrazine derivatives on bacterial communities inhabiting chicken eggs. The utilization of next-generation sequencing and corresponding bioinformatics analyses showed a high diversity on order level in the samples. Some orders, such as *Pseudomonadales* and *Clostridiales* were prominent in most of the samples. Pyrazine treatments were not followed by reoccurring community changes on order level when samples from different sources were compared. Interestingly, the non-template control (WA_c) showed similar order distributions like the samples. This is a known phenomenon in samples with low DNA content due to sample bleeding when an Illumina platform is used^29^. On phylum level the composition of the untreated samples and the active treated samples were very similar. In contrast, the passive treatment resulted in a different community composition and showed an increase in *Proteobacteria*. Passive treatment as shown in the growth dependent methods was not able to sufficiently reduce the high microbial count on the egg surfaces. The amplicon sequencing data indicate a clear effect on the structure of the microbiome. Passive treatment induced a community change due to lower pyrazine concentrations. Such community changes are common when only a distinct fraction of the present microorganisms is susceptible to an introduced substance. Antibiotics are prominent causes for global microbiome shifts that can have drastic effects on the integrity of the microbial population^30^.

*Micrococcus* was found to be the most prominent genus with significantly differing occurrence in the pyrazine-treated and control samples. It is known from former studies that *Micrococcus* among a broad number of *Enterobacteriacea* is widely found on egg samples^18,31^. The treatment efficiency of this prevalent colonizer with the utilized pyrazine derivative was highly promising. *Micrococcus* spp. are linked to embryonic death and reduced hatchability in hatcheries^32^. All other OTUs that were differentially affected by the treatment had a more than 2-fold lower occurrence than *Micrococcus*. While most of the identified bacteria had the highest occurrence in the not treated samples, some increases of distinct OTUs were also observed during passive treatment. These OTUs included potential pathogens such as *Clostridiales* or *Pseudomonadaceae*. *Clostridiales* were also found to be increased in some active treated samples on order level. However, this was not confirmed by the differential occurrence analysis. Therefore, the unambiguous effect of alkylated pyrazines on them remains to be elucidated in detail. The enrichment of specific OTUs during the treatment can influence the final composition of the microorganisms colonizing the hatching eggs during the incubation. This can subsequently have drastic consequences on the development and health of the hatched chickens. In light of this, other studies described differing formaldehyde susceptibility of various bacteria due to present detoxification mechanisms. Bacteria that were found to employ efficient formaldehyde detoxification mechanisms include pathogenic strains of *Escherichia coli*, *Klebsiella pneumonia*e, *Listeria monocytogenes*, *Pseudomonas aeruginosa*, and *Staphylococcus aureus* ^33^. It can be expected that fumigation of these and other resistant microorganisms within a complex community would cause a measureable shift. This remains to be explored within complementary studies. We see high potential to further improve the utilization of alkylated pyrazines for disinfection purposes of hatcheries and other industrial facilities. Moreover, this study provides a good framework for further developments of more sustainable eggshell treatments in industrial hatcheries.

## Material and Methods

### Diazine treatments of eggshells

Egg samples for this study were obtained from four different sources. Two suppliers from the supermarket, eggs from the farmers market, and eggs from a farm-gate sale (Styria/Austria) were analyzed within this study. Table 1 shows detailed specifications of the analyzed egg samples. Two different diazine treatments were evaluated in the frame of this study. For the passive treatment, 12 eggs from each producer were incubated in a closed plastic container. A total of 10 mL of 5-isobutyl-2,3-dimethylpyrazine (Sigma-Aldrich/Germany) was placed in a petri dish underneath the eggs. During a 6 h incubation time the pyrazine evaporated partially into the headspace surrounding the eggs. As a control, eggs were incubated using the same plastic containers without addition of pyrazine. The active treatment was performed in a glass desiccator. A total of 1 mL of 5-isobutyl-2,3-dimethylpyrazine was evaporated underneath the eggs using a heating plate set on 45 °C. The heating plate did not lead to a significant increase of the temperature. The eggs were incubated for 6 h. As a control, eggs were incubated for the same time without addition of pyrazine.

**Table 1:** Sample description of the utilized chicken eggs in this study.

Sample abbreviation	Sample origin	Farming method	Approval number available
TF	Supermarket	Free range	Yes
DB	Supermarket	Deep litter system	Yes
PR	Farmers market	Free range, organic	Yes
AZ	Farm-gate sale	Free range	No

### Processing of eggshells after the treatments

Egg samples were prepared with a standardized protocol to analyze contamination levels on the treated samples compared to the control samples. Cultivation dependent methods, real-time qPCR, as well as amplicon sequencing of the 16 S rRNA gene fragment were performed using the same samples. For the detachment of surface microorganisms, the shells from three pooled eggs were washed for 15 min at 400 rpm with 20 mL of PBS in 50 ml reaction tubes. Aliquots of the obtained suspension were utilized for further analyses. Samples for molecular studies were treated with propidium monoazide (PMA) to allow discrimination between living and dead cells. A total of 1 mL of the previously prepared egg shell suspension was treated with 20 µL PMA to reduce the free DNA or DNA from dead bacteria in the sample^33^. After adding the PMA, the samples were incubated for 50 min in the dark with slight shaking every 10 min in 1.5 ml reaction tubes. To start the PMA reaction, the samples were placed under blue LED light (465–475 nm) for 10 min with opened lids. After PMA treatment, the suspension was centrifuged at 16000 rpm for 20 min and 4 °C. The pellet was further used for total DNA extraction and 16 S rRNA gene-based analyses. For each producer and treatment four samples and four untreated controls were obtained.

Genomic DNA was extracted from the samples using the FastDNA® Kit for Soil (MP Biomedicals/USA). In the first step, the pellet was resuspended in 978 µL Sodium Phosphate buffer. Other steps were conducted according to the manufacturer’s instructions.

### Cultivation-based quantification of bacteria on egg shells

Following the washing step, 100 µL of undiluted suspension, a 10^−1^, and a 10^−2^ dilution were plated on nutrient agar (Carl Roth/Germany) to quantify colony forming units (cfu). Following incubation of the agar plates over night at 30 °C, the cfu were counted on evaluable plates. Lower cfu counts indicated a reduced contamination level in the sample.

### RT qPCR-based quantification of bacteria on egg shells

Total DNA extracts from the egg samples were further analyzed using qPCR. The analysis was performed using a real-time quantitative method. Gene copy numbers of the 16 S rRNA gene fragment were obtained using the Unibac II 515 f and 927r primer pair and the corresponding temperature program^34^. The quantification was performed with a Corbett Research TM thermocycler (Rotor-Gene 6000, Corbett Research/United Kingdom) and SYBR Green PCR master mix TM (KAPA Biosystems/USA). The standard curve was obtained using a *Bacillus cereus* 16 S rRNA gene fragment and further 1:10 dilutions. Three replicates of each standard dilution were prepared to generate a mean value. The standard values were employed later on to determine the gene copy numbers in the analyzed samples. Negative controls (pure dH_2_O was added to the DNA extraction kit) were also analyzed and further subtracted from the analyzed sample values to reduce method-derived inaccuracies. All analyses were repeated three times and used to calculate mean values.

### Barcoding of amplicon samples

The extracted DNA was used as a template for PCR amplification. Barcoded PCR was performed using the universal bacterial primer set 515 f/806r to target the 16 S rDNA hypervariable region 4^35^. The PCR step for each sample was performed four times and after performing a control gel to identify the correct PCR fragment length, the amplified DNA was purified and subsequently pooled using the Wizard® SVGel and PCR Clean Up System Kit (Promega/Germany). Barcoded samples were pooled equimolarly and sent for paired-end HiSeq Illumina sequencing (GATC Biotech/Germany).

### Structural community analysis with 16S rRNA gene amplicons

After HiSeq Illumina sequencing of the barcoded PCR fragments the data was analyzed using the QIIME 1.9.0 pipeline^36,37^. Initially, the raw Illumina HiSeq forward and reverse reads were joined (default method: fastq-join) for all of the 64 samples. The fastq sequence data demultiplexing was performed with QIIME default setting. Additionally, the sequences were quality-checked for chimeric sequences (usearch7; Edgar, 2010). Subsequently, the OTU table was generated with the script “pick_open_reference_otus.py” using default settings and reference database. Single- and doubletons were filtered from the dataset. The greengenes database (release 13_5) was employed for reference sequences and taxonomy assignment. OTU clustering was performed with a sequence similarity threshold of 97% representing theoretical taxonomic units at species level. The “core_diversity_analyses.py” script was used to generate all summarize taxa through plots, alpha- and beta-diversity plots as well as statistical evaluations plugging several scripts together. Sampling depth was rarefied to 21,000 based on the biome summarize-table outcome and the lowest number of counts. Comparative analysis on OTU frequencies to identify statistically significant differences between OTU abundances in the three different treatments was performed with the QIIME script “group_significance.py” using the Kruskal-Wallis test based on the rarified OTU table. Based on the comparative analysis a pie-charted network was generated using Cytoscape 3.4.0 to visualize the significant differences between the treatments. The cutoff for deepening analyses was placed after the first 100 OTUs with the lowest p-value.

### Differential *in situ* visualization of bacterial contaminations

Fragmented eggshells from treated and control samples were stained with the LIVE/DEAD® Baclight kitTM (Molecular Probes). The imaging was performed with a confocal laser scanning microscope (Leica TCS SPE confocal microscope, Leica Microsystems). Excitation wavelengths of 488 and 532 nm were used for the SYTO® 9 and propidium iodide fluorescent dye respectively. The light emission was detected in the range of 496–560 nm for SYTO® 9 and 600–680 nm for propidium iodide. Settings for photomultiplier gain and offset were adjusted to obtain an optimal signal/noise ratio. The confocal stacks were merged to obtain a maxium projection of all channels.

### Statistical analysis

For cultivation-dependent and qPCR-based experiments, the statistical analysis was performed using Student’s paired t-test. Statistical analyses of the amplicon data were performed within the QIIME 1.9.0 pipeline^36,37^.

### Data availability

All publication related amplicon data is available at ENA: study accession number PRJEB21532.

## Electronic supplementary material

Supplementary Dataset 1
