# Supplementary material for: Replacing conventional decontamination of hatching eggs with a natural defense strategy based on antimicrobial, volatile pyrazines
Source: Sci Rep. 2017 Oct 16;7:13253. doi: 10.1038/s41598-017-13579-7 (PMC5643471; doi:10.1038/s41598-017-13579-7)
Supplement: Supplementary file 1 — Supplementary Dataset 1 [file 41598_2017_13579_MOESM1_ESM.doc]

**Replacing conventional decontamination of hatching eggs with a natural defense strategy based on antimicrobial, volatile pyrazines**

**Peter Kusstatscher^1,2#^, Tomislav Cernava^1,2*#^, Stefan Liebminger^3^, and Gabriele Berg^2^**

*^1^ACIB GmbH, Petersgasse 14, 8010 Graz, Austria*

*^2^Institute of Environmental Biotechnology, Graz University of Technology, Petersgasse 12, 8010*

*^3^Roombiotic GmbH, Petersgasse 12, 8010 Graz, Austria*

^#^Both authors contributed equally

**Correspondence:*

*Tomislav Cernava, Institute of Environmental Biotechnology,*

*Graz University of Technology, Petersgasse 12,*

*8010 Graz, Austria.*

*e-mail:* [*tomislav.cernava@tugraz.at*](mailto:gabriele.berg@tugraz.at)

**Submitted to:** Scientific Reports

**Supplementary data**


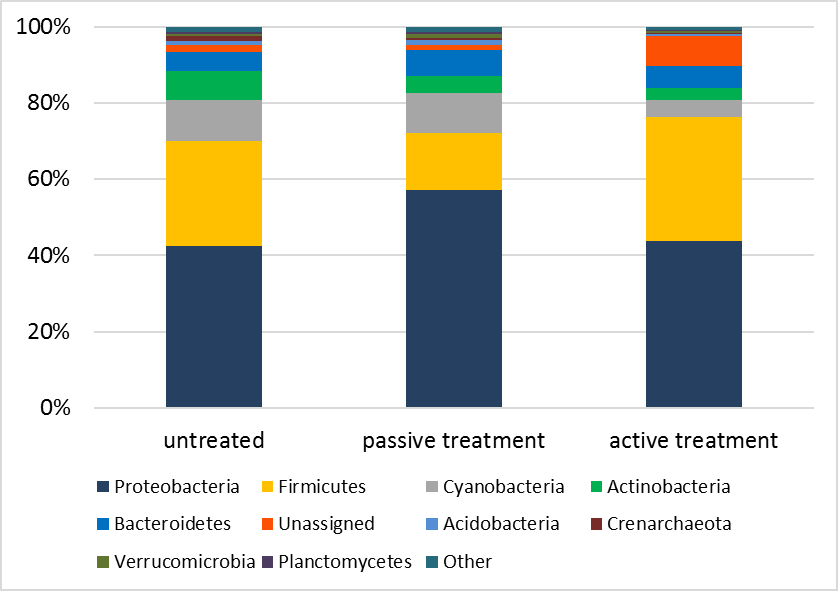


**Figure S1:** Bacterial community on treated and untreated egg samples based on amplicon data. Identified OTUs are shown on phylum level.
